# Straight to Phase III: Model‐Informed Approach Speeds Depemokimab Clinical Development in Interleukin‐5‐Driven Diseases

**DOI:** 10.1002/cpt.70183

**Published:** 2026-01-20

**Authors:** Chiara Zecchin, Stein Schalkwijk, Isabelle J. Pouliquen, Alienor Berges, Nicholas Bird, Richard Follows, Daren Austin

**Affiliations:** ^1^ Clinical Pharmacology Modelling and Simulation GSK Stevenage UK; ^2^ GSK Amsterdam The Netherlands; ^3^ Clinical Pharmacology Modelling and Simulation GSK London UK; ^4^ Clinical Pharmacology GSK Stevenage UK; ^5^ Biostatistics GSK London UK; ^6^ Clinical Sciences – Respiratory Immunology & Inflammation Unit (RIIRU), GSK London UK; ^7^ Business Development GSK London UK

## Abstract

IL‐5, a key mediator of type 2 inflammation, underlies various diseases, including severe asthma, CRSwNP, EGPA, and HES. Reduction in blood eosinophil count (BEC), a biomarker of IL‐5 activity, is commonly used to evaluate the efficacy of anti‐IL‐5 biologic therapies. Model‐informed drug development (MIDD) and quantitative decision making (QDM) were used to shorten the clinical development of depemokimab (an ultra‐long‐acting anti‐IL‐5 biologic). A Bayesian nonlinear mixed effects dose–time response model predicted the depemokimab dose in severe asthma achieving comparable BEC reductions to those observed in mepolizumab (an approved anti‐IL‐5 biologic) Phase III MUSCA and MENSA trials. Prespecified QDM go/no‐go criteria were applied to assess success probability. Phase IIb efficacy‐based trial simulations were conducted using negative binomial distribution to simulate individual annualized exacerbation rate. A depemokimab PK/PD (BEC) model predicted Phase III trial doses in CRSwNP/EGPA/HES. Single depemokimab doses were well‐described by the Bayesian model; a single depemokimab dose ≥ 60 mg had probability ≥ 80% of exceeding Minimum (78%; MUSCA) and ≥ 10% probability of exceeding Target (84%; MENSA) values for trough BEC reduction from baseline vs. placebo. Clinical trial simulations demonstrated < 3% probability of more precise estimation of the Phase III dosing regimen with a conventional efficacy‐based dose‐ranging study. Depemokimab 100 mg for severe asthma/CRSwNP and 200 mg for EGPA/HES, administered subcutaneously every 26 weeks, were selected for Phase III trials. MIDD and QDM shortened the depemokimab development program by 2–3 years, emphasizing the potential of this approach for progressing new therapies from Phase I directly to Phase III.

## Graphical abstract

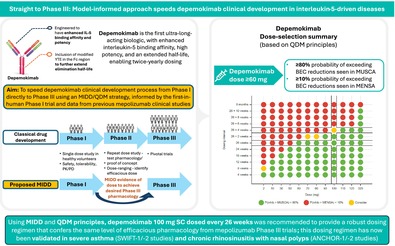

**Study Highlights**

**WHAT IS THE CURRENT KNOWLEDGE ON THE TOPIC?**

Current anti‐IL‐5 therapies, such as mepolizumab and other approved biologics, effectively reduce BEC with dosing every 4–8 weeks, which translates into clinical efficacy.

**WHAT QUESTION DID THIS STUDY ADDRESS?**

This work outlines the significantly shortened clinical development pathway of depemokimab, an ultra‐long‐acting anti‐IL‐5 biologic for diseases with underlying type 2 inflammation, that potentially enables sustained efficacy with less frequent twice‐yearly dosing, achieved by skipping a Phase II dose‐ranging trial through the use of model‐informed drug development (MIDD) and quantitative decision making (QDM).

**WHAT DOES THIS STUDY ADD TO OUR KNOWLEDGE?**

This study demonstrates the novel application of MIDD and QDM to enable dose selection with enhanced precision and confidence, streamlining the drug development process.

**HOW MIGHT THIS CHANGE CLINICAL PHARMACOLOGY OR TRANSLATIONAL SCIENCE?**

MIDD and QDM shortened the depemokimab development program by approximately 2–3 years and successfully predicted an appropriate therapeutic dose based on established same‐class results from Phase III trials, supporting the potential of this approach to accelerate drug development.

The role of interleukin (IL)‐5 in type 2 inflammation has been a major focus of research in the past decades, highlighted by the efficacy of IL‐5‐targeted therapies in various disease states.^1^ Mepolizumab is an immunoglobulin‐G1 monoclonal antibody that neutralizes dimeric IL‐5,^2^, ^3^ and produces a sustained, dose‐dependent reduction in blood eosinophil count (BEC; a biomarker of type 2 inflammation).^4^ Mepolizumab has demonstrated good clinical efficacy in various IL‐5‐driven diseases^5^, ^6^, ^7^, ^8^, ^9^ and was approved in 2015 as the first anti‐IL‐5 biologic treatment for severe asthma with an eosinophilic phenotype (100 mg dose administered subcutaneously [SC] every 4 weeks [Q4W]).^2^, ^3^ It was subsequently approved for use in eosinophilic granulomatosis with polyangiitis (EGPA; 300 mg SC Q4W) in 2017, hypereosinophilic syndrome (HES; 300 mg SC Q4W) in 2020, and chronic rhinosinusitis with nasal polyps (CRSwNP; 100 mg SC Q4W) in 2021.^2^, ^3^ Mepolizumab 100 mg SC reduced BEC in patients with severe asthma from baseline by 84% and 78% compared with placebo in the Phase III MENSA and MUSCA studies, respectively,^6^, ^9^ and was associated with substantial BEC reductions (81–92%) in patients with EGPA, HES, or CRSwNP.^5^, ^8^, ^10^ Efficacy dose–response relationships between BEC reduction and efficacy have not been forthcoming. However, the BEC reductions described above provide precedented targets for establishing clinical benefit in IL‐5‐driven diseases.^5^, ^6^, ^7^, ^8^, ^9^ Beyond the pharmacodynamic (PD) effect of mepolizumab on eosinophils, which has been shown to translate into clinical efficacy,^6^, ^11^ mepolizumab and its associated neutralizing action on IL‐5 are associated with a favorable safety profile based on thousands of patient‐years of exposure.^12^, ^13^, ^14^

Depemokimab is the first ultra‐long‐acting anti‐IL‐5 biologic engineered with enhanced binding affinity, high potency, and an extended half‐life, offering the potential for twice‐yearly dosing for patients and sustained suppression of type 2 inflammation.^15^, ^16^ Importantly, depemokimab and mepolizumab are distinct antibodies: while both target the same IL‐5 epitope, depemokimab differs from mepolizumab in terms of changes to the heavy chain variable region and the Fc region. Specifically, four amino acid changes were introduced in the heavy‐chain variable region and three additional changes were made in the Fc region (known as the YTE modification). These modifications to the heavy‐chain variable and Fc regions of depemokimab, respectively, increase affinity for IL‐5 (thus conferring higher potency) and extend depemokimab’s half‐life.^17^ These differences between depemokimab and mepolizumab were expected to confer a longer duration of action without affecting the clinical efficacy and safety profiles.

The safety, tolerability, pharmacokinetic (PK), and PD effects of depemokimab on BEC were evaluated in a first‐time‐in‐human (FTiH), single‐ascending‐dose Phase I trial.^18^ The trial included patients with mild‐to‐moderate asthma and BEC ≥ 200 cells/μL at screening; the latter serving as an indicator of type 2 inflammation, with the aim of permitting translation to a broader type 2 patient population. Trial results indicated that depemokimab was well‐tolerated and had a linear dose‐proportional PK with a geometric mean half‐life ranging from 38 to 53 days (approximately twice that of mepolizumab). A reduction in BEC was seen with all depemokimab doses investigated from the first post‐dose assessment (Day 2; 24 hours), with a clear dose–response observed for the return toward baseline, and little change post dose in the placebo group.^18^

Typically, following Phase I single‐ascending‐dose testing, asset clinical development would proceed to multiple‐ascending‐dose testing and then Phase II repeat‐dose/dose‐ranging studies to establish proof of concept and identify optimal dose regimen. Model‐informed drug development (MIDD) is a complementary methodology that has the potential to streamline and consequently accelerate the development of new medicines by enhancing/enabling informed decision making.^19^ MIDD is a quantitative framework that uses mathematical and statistical models to support drug development and regulatory decision making by integrating diverse preclinical, clinical, and real‐world data to simulate and predict the behavior of a drug in various scenarios.^19^, ^20^ In the context of new drug development, MIDD can be used to aid the design of clinical trials, support the selection of the sample size, trial duration, and optimal dose, and is increasingly being used to predict drug–drug interactions, assist formulation development, and to guide strategy from in vivo studies to post‐approval lifecycle plans.^19^, ^21^ In the future, MIDD may also support the development of cell and gene therapy products.^19^ Recently, the MIDD approach has been formalized by the US Food and Drug Administration and European Medicines Agency.^22^ The International Council for Harmonisation of Technical Requirements for Pharmaceuticals for Human Use has also published draft M15 guidance on general principles for MIDD.^23^

Quantitative decision making (QDM) is a formal decision‐making strategy that supports progression of drug development and is routinely applied in this process. QDM predicts the probability of future outcomes based on current data and uses formal go and no‐go decisions based on the probability of attaining a desired outcome range for an endpoint (e.g., efficacy or pharmacology endpoints).^24^ Where outcomes are to be either interpolated or extrapolated from current data (e.g., a different dose, regimen, or patient population), MIDD may be used to predict the probability of success of various untested scenarios. In the Phase I depemokimab trial, MIDD was integrated into the QDM framework; it was anticipated that the application of both QDM and MIDD would improve the quality, efficiency, and cost effectiveness of decision making.

This article describes the process of speeding clinical development for depemokimab by moving directly from Phase I to Phase III using a MIDD/QDM strategy, informed by the Phase I FTiH asthma trial data from previous mepolizumab clinical studies, and clinical trial simulations. The results of this process enabled confident dose selection for Phase III depemokimab registration studies across various indications without the need for conventional Phase II proof‐of‐concept/dose‐ranging studies.

## METHODS

### Data sources

The MIDD/QDM strategy was informed by data from the Phase I FTiH asthma trial of patients with mild‐to‐moderate asthma receiving depemokimab,^18^ two Phase III mepolizumab clinical trials in patients with severe asthma (MENSA and MUSCA),^6^, ^9^ CRSwNP (SYNAPSE),^5^ HES (study 200862),^25^ and EGPA (study MEA115588),^26^ trial simulation using Phase IIb mepolizumab exacerbation data,^7^ and a population PK/PD meta‐analysis of data from 16 mepolizumab trials of participants with various eosinophilic conditions and healthy participants treated with mepolizumab.^5^, ^8^, ^10^, ^25^, ^26^, ^27^, ^28^, ^29^, ^30^, ^31^, ^32^, ^33^, ^34^, ^35^, ^36^, ^37^ Patients in the Phase I FTiH asthma trial were ≥ 18 years of age and had mild‐to‐moderate asthma, a BEC ≥ 200 cells/μL at screening, and were being treated with low‐to‐moderate inhaled corticosteroid (ICS) or ICS/long‐acting β_2_‐agonist therapy.^18^ Patients included in the MENSA and MUSCA studies were ≥ 12 years of age with severe asthma, had ≥ 2 exacerbations in the prior year requiring treatment with systemic corticosteroids despite high‐dose ICS treatment and additional controllers, and a BEC ≥ 150 cells/μL at screening or ≥ 300 cells/μL in the prior year.^6^, ^9^ The 16 studies in the mepolizumab population PK/PD meta‐analysis included participants with a variety of BEC at baseline, diseases, demographic characteristics, and dosing regimens.^5^, ^8^, ^10^, ^25^, ^26^, ^27^, ^28^, ^29^, ^30^, ^31^, ^32^, ^33^, ^34^, ^35^, ^36^, ^37^

### Modeling the dose–time response for depemokimab based on Phase I data

BEC data from the depemokimab Phase I FTiH asthma trial (post‐Day 28) were used to develop a Bayesian nonlinear mixed effects dose–time response model, with covariates of baseline BEC and time,^18^ to characterize BEC suppression and to simulate the expected PD response in Phase III. The model included random effects for the intercept of placebo and active treatment, maximum effect (*E*_max_), and log‐half maximum dose (LNED_50_).

The Bayesian analyses were conducted using the Statistical Analysis System procedure for Bayesian analysis, using Markov Chain Monte Carlo methods. Model convergence and robustness were evaluated using multiple diagnostics, including the Geweke, Heidelberger–Welch, and Raftery–Lewis Diagnostic, along with inspection of trace and density plots. Model convergence was satisfactory across all criteria (data not shown).

### Selecting Phase III depemokimab dose in patients with severe asthma using QDM principles

The depemokimab Phase III dose was selected using a pharmacology matching approach. The precise depemokimab dose needed to provide reductions in blood eosinophil pharmacology comparable to those seen with mepolizumab was identified using the Bayesian dose–time response model developed based on depemokimab Phase I data.^18^ It was expected that matching or exceeding established BEC reductions would translate into comparable/precedented clinical efficacy, despite the lack of an established relationship between BEC reduction on‐treatment and efficacy. The BEC reductions achieved in the positive mepolizumab Phase III MENSA and MUSCA studies were used as prespecified QDM go and no‐go criteria and applied to dose–dosing regimen combinations in accordance with the PSI special interest group working group on QDM.^38^, ^39^, ^40^ A regimen was deemed a “go” if the probability of exceeding the Minimum Value (MV) was > 80%, and a regimen was deemed a “no go” if the probability of exceeding the Target Value (TV) was < 10%, with the MV and TV defined by the 78% and 84% BEC reductions vs. placebo observed in MUSCA and MENSA, respectively. Anything falling between these values was deemed a “consider,” in accordance with QDM principles.^39^ The probability thresholds in QDM are grounded in decision theory, risk tolerance, and industry best practices, consistent with published frameworks.^38^, ^40^ It was anticipated that MIDD could extrapolate depemokimab pharmacology for new doses, dosing frequencies, and/or diseases using in silico data.

The Bayesian dose–time response model was used to simulate log‐change from placebo predictions in BEC for 12 depemokimab doses (2–300 mg SC) and 12 sampling times (Days 28–280 post dose at approximately 4‐week intervals) that were tested against the log BEC ratios sampled from the MUSCA and MENSA trials (MV and TV, respectively) for BEC reduction.^6^, ^9^ Probabilities were calculated from each of 10,000 Markov Chain Monte Carlo simulations and plotted graphically.

### Simulating Phase IIb dose‐ranging studies in severe asthma

The aim of this modeling exercise was to assess whether conducting a conventional Phase IIb trial powered to detect a dose–response relationship using a clinical efficacy endpoint (asthma exacerbation) would provide better precision and confidence to inform Phase III dose selection compared with the pharmacology approach outlined above based on BEC dose–response. To determine the statistical power and precision needed to detect an exacerbation effect and a dose–response relationship with active treatment vs. placebo in patients with severe asthma, clinical trial simulations of Phase IIb trial designs were conducted, for an unknown dose–response to placebo, and between two and five active doses per trial (selected from the following 15 possible doses ranging from 2 to 300 mg). Using combinatoric selection, for every complete set of dose combinations (i.e., all possible 2, 3, 4, and 5‐arm trial active dose choices), 3000 trial designs were selected at random with replacement and a placebo treatment added. Individual trial parameters were also selected at random to propagate parameter uncertainty in half‐maximal dose (ED_50_). Individual annualized exacerbation rates were simulated using a negative binomial distribution for sample sizes of 60 to 600 patients (with 10–200 per treatment arm, and only balanced treatment arm sample sizes considered). BEC at baseline, *E*_max_ and dispersion (*k*) were based on information from mepolizumab Phase III studies with an unknown dose–response location (ED_50_). The simulated trials were fitted using two models: (1) a generalized linear model with treatment as a class effect, and (2) a nonlinear dose–response model with dose as a continuous variable. The ED_90_ (defined as the effective dose that achieves a population average of 90% of the maximum response) was estimated as the desired future clinical dose. The power to detect a treatment effect and median estimated clinical dose were summarized by the number of active doses and the population sample size.

### Dose selection in CRSwNP, EGPA, and HES based on BEC reduction

To identify the depemokimab doses that confer the same level of BEC reduction as seen with mepolizumab in patients with CRSwNP, EGPA, and HES, further modeling was conducted to bridge from the depemokimab FTiH asthma study to these indications. Unlike in asthma, where the available Phase I data supported an empirical dose–response approach to describe BEC suppression, extrapolation to CRSwNP, EGPA, and HES required a more mechanistic framework. This was necessary to account for disease‐specific differences in pathophysiology and blood eosinophil kinetics that may influence PD response. Accordingly, a mechanistic model informed by mepolizumab data, where clinical experience exists across these eosinophilic diseases, was used to contextualize depemokimab’s expected effects and support dose selection beyond the asthma population. A linear one‐compartment PK model with first‐order absorption coupled to an indirect exposure–BEC model was used. The indirect exposure‐BEC model was parameterized with terms for baseline BEC (KRO), rate of elimination (KOUT), maximum inhibitory effect (*I*_max_), concentration resulting in 50% of maximum inhibitory effect (IC_50_), and Hill coefficient (GAMA) (refer to the equation below). Between‐patient variability for baseline BEC and maximum inhibitory effect were included using an exponential model, and measured baseline BEC was a covariate of KRO and *I*_max_. Disease population was also a covariate for KRO. The model IC_50_ parameter, between‐patient variability, and the residual unexplained variability were estimated based on the PK and blood eosinophil data from the Phase I depemokimab trial,^18^ with all other parameters and model structure fixed to those estimated from the population PK/PD meta‐analysis of pooled data from 16 mepolizumab studies.^5^, ^8^, ^10^, ^25^, ^26^, ^27^, ^28^, ^29^, ^30^, ^31^, ^32^, ^33^, ^34^, ^35^, ^36^, ^37^ The exposure–BEC model had been validated previously and refined further using mepolizumab data from Phase III trials in CRSwNP, EGPA, and HES.^5^, ^8^, ^10^

$$\frac{\text{deos}\left(t\right)}{\mathrm{dt}}=\text{KOUT}\cdot \left(\mathrm{KRO}\cdot \left(1+\left(I_{\max}-1\right)\cdot \frac{C{\left(t\right)}^{\text{GAMA}}}{C{\left(t\right)}^{\text{GAMA}}+{\mathrm{IC}}_{50}^{\text{GAMA}}}\right)-\mathrm{eos}\left(t\right)\right)$$

Several dose regimens were simulated, with 1000 simulations for each dose regimen in each disease population (CRSwNP, EGPA, or HES). Baseline BECs were set to those in the respective Phase III mepolizumab studies for each disease. Additional simulations for EGPA focused on baseline BEC of 1000 cells/μL, which is aligned with the criteria for diagnosis of untreated EGPA,^41^ and is considered more indicative of “true” baseline BEC when oral corticosteroids (OCS) are tapered to achieve efficacy criteria. The dose regimen that provided the target pharmacology range to confer the same level of BEC reduction as seen with mepolizumab for the majority of the duration of the trial was selected for each disease population.

### Informed consent and ethics

Ethics approval was not required for this study as it involved the analysis of existing, anonymized data and did not include any direct patient involvement or intervention; consequently, informed consent was not applicable. The source studies for data used in these analyses received approval from the appropriate ethics committees; all data in those studies were from patients who had provided informed consent.

## RESULTS

### Model development based on BEC reduction in severe asthma from depemokimab Phase I FTiH asthma data

**Figure 1:**
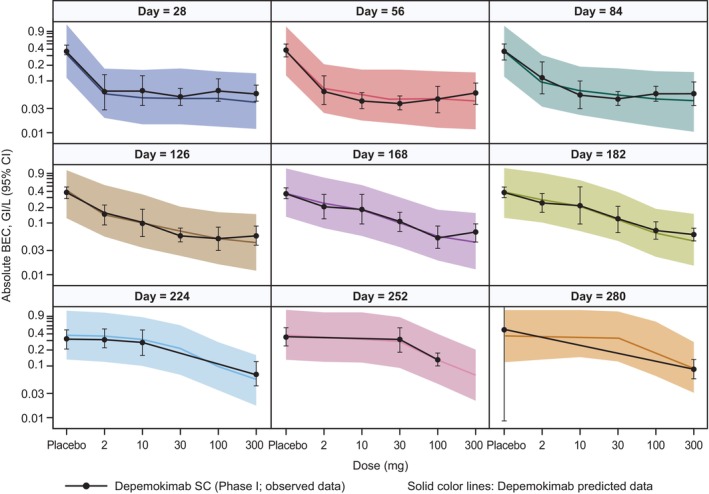
Absolute BEC dose–response by time. BECs from a Phase I study of depemokimab in patients with mild‐to‐moderate asthma were modeled using a Bayesian nonlinear mixed effects dose–time response model. The black line is the mean (95% CI) observed data from the Phase I trial and the colored plain line is the predicted median from the model. The colored shaded areas represent 95% CrI. BEC, blood eosinophil count; CI, confidence interval; CrI, credibility intervals.

The Bayesian dose–time response model to guide depemokimab dose selection for severe asthma, based on BEC reduction, was informed by 622 blood eosinophil measurements from 48 patients with mild‐to‐moderate asthma in the depemokimab Phase I FTiH asthma trial. Of these, only measurements collected from Day 29 post depemokimab administration onward were used to characterize the return to baseline BEC, where a clear dose–response relationship was observed, with 293 observations (from Day 57 onward) used for model development and 47 observations (Day 29) used for model validation. The final model parameters are shown in **Table** **1**. The Bayesian nonlinear mixed effects dose–time response model provided an excellent description of the BEC return to baseline data (i.e., Day 57 onward) for all patients at all doses and times, and also accurately predicted Day 29 data not included in the analysis, thus validating the model (**Figure** **1**).

**Table 1:** Final dose–time response parameter estimates

Parameter	*N*	Mean (SD)	Median	Highest posterior density (lower, upper)
E0	10,000	−0.2601 (0.3054)	−0.2640	−0.8438, 0.3425
E1	10,000	−0.6583 (0.4209)	−0.6550	−1.5362, 0.1422
E2	10,000	0.5128 (0.1995)	0.5084	0.1373, 0.9072
E3	10,000	0.5084 (0.3865)	0.3425	−0.5108, 1.0546
*E*_max_	10,000	−2.6047 (0.2451)	−2.6089	−3.0449, −2.1338
LNED_50_ (Day 182)	10,000	2.9513 (0.2093)	2.9562	2.5644, 3.3695
ED_50_ mg (Day 182)	NA	19.1308 (NA)	19.2248	12.9929, 29.0640
KE (/day)	10,000	0.0207 (0.00263)	0.0204	0.0159, 0.0259
T_1/2_ (days)	NA	33.4854 (NA)	33.8778	43.5942, 26.7624^a^
Hill slope	10,000	1.2358 (0.2222)	1.2215	0.8271, 1.6974
F (2 mg dose)	10,000	2.3730 (0.9708)	2.2405	0.6863, 4.2986
S^2^B_1_ (E0)	10,000	0.2096 (0.0770)	0.1961	0.0870, 0.3632
S^2^B_1_B_2_	10,000	−0.2058 (0.1034)	−0.1888	−0.4173, −0.0305
S^2^B_1_B_3_	10,000	0.0550 (0.0795)	0.0560	−0.1114, 0.2108
S^2^B_2_B_1_	10,000	−0.2058 (0.1034)	−0.1888	−0.4173, −0.0305
S^2^B_2_ (*E*_max_)	10,000	0.5194 (0.1881)	0.4908	0.2033, 0.8942
S^2^B_2_B_3_	10,000	−0.2120 (0.1462)	−0.1884	−0.5221, 0.0461
S^2^B_3_B_1_	10,000	0.0550 (0.0795)	0.0560	−0.1114, 0.2108
S^2^B_3_B_2_	10,000	−0.2120 (0.1462)	−0.1884	−0.5221, 0.0461
S^2^B_3_ (LNED_50_)	10,000	0.3850 (0.2205)	0.3317	0.0897, 0.8094
S^2^Y (EPS)	10,000	0.0804 (0.00787)	0.0800	0.0654, 0.0959
**Model** LNEOS = b1 + E0 + E2*LNBASE + (*E*_max_ + b2 – E0) /(1 + exp(SLP*(b3 + LNED_50_ + KE*(TDAYS‐182) ‐ log(F) ‐ LNDOSE))) + EPS (for depemokimab treatment) LNEOS = b1 + E1 + E3*LNBASE + EPS (for placebo) **Between‐patient variability** BPV = SQRT(exp(S^2^B_1_) – 1) = 48.3% BPV = SQRT(exp(S^2^B_2_) – 1) = 82.5% BPV = SQRT(exp(S^2^B_3_) – 1) = 68.5% **Residual error** BOV = SQRT(exp(EPS) – 1) = 28.9%				

BEC, blood eosinophil count; BOV, between‐occasion variability; BPV, between‐patient variability; ED_50_, exp(LNED_50_); *E*_max_, maximum effect; E0, E1, baseline log‐BEC; EPS, residual variance; *F*, relative bioavailability of 2 mg dose; LNBASE, log(Baseline); LNED_50_, log(half‐maximal dose on d182); KE, rate of increase of ED_50_; SLP, Hill slope; LNDOSE, log(DOSE); NA, not applicable; S^2^B_n_, between‐patient baseline variance; SD standard deviation.

^a^ The values for half‐life credibility interval were derived directly from the estimates of the elimination rate constant (KE). Because half‐life is inversely related to KE (i.e., a smaller rate corresponds to a longer half‐life and vice versa), the numerical order of the interval appears reversed. The values were retained as calculated to preserve consistency with the underlying KE estimates.

### Simulations for Phase III depemokimab dose selection in patients with severe asthma coupled with use of QDM principles

The Bayesian dose–time response model was used to simulate a range of depemokimab doses at different timepoints, and QDM principles were applied to identify the Phase III dose that would achieve a comparable level of blood eosinophil reduction from baseline vs. placebo as seen with mepolizumab 100 mg SC in patients with severe asthma in the MENSA and MUSCA trials.^6^, ^9^

**Figure 2:**
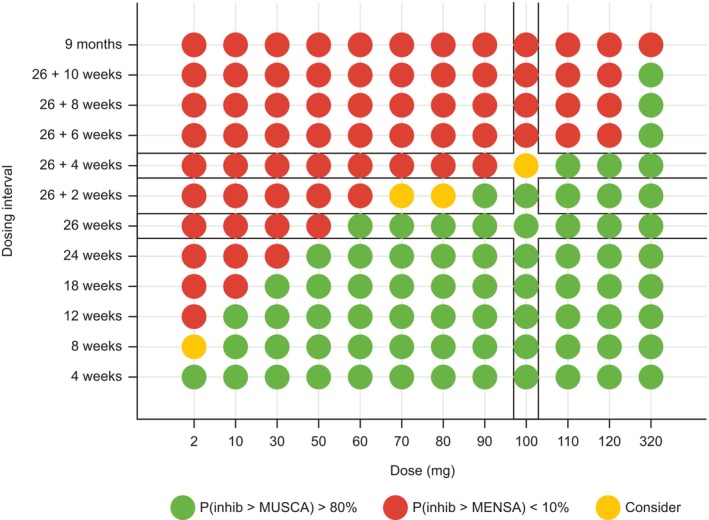
Probability of exceeding the MENSA and MUSCA pharmacology based on QDM principles. Prespecified QDM go and no‐go criteria were applied to the 12 doses and 12 dosing intervals. A regimen was deemed a “go” provided the probability of exceeding the MV was > 80% (green), and a regimen was deemed a “no go” if the probability of exceeding the TV was < 10% (red). Anything falling between these values was deemed a “consider” (yellow). MV, minimum value; QDM, quantitative decision making; TV, target value.

Using QDM principles, comparable BEC reductions from baseline between depemokimab and mepolizumab at 26 weeks post dose indicated that a single depemokimab dose ≥ 60 mg had ≥ 80% probability of exceeding MV and ≥ 10% probability of exceeding the TV observed with mepolizumab in MUSCA and MENSA, respectively (**Figure** **2**, **Figure** **S1**). Thus, depemokimab 100 mg SC dosed every 26 weeks (Q26W) was recommended with high precision to provide a robust dosing regimen that was expected to be comparable to the efficacious pharmacology of mepolizumab from Phase III trials, while permitting some flexibility in the precise dosing interval to allow for more practical dosing visits (of up to an additional 4 weeks).

### Simulating Phase IIb dose‐ranging studies in severe asthma

**Figure 3:**
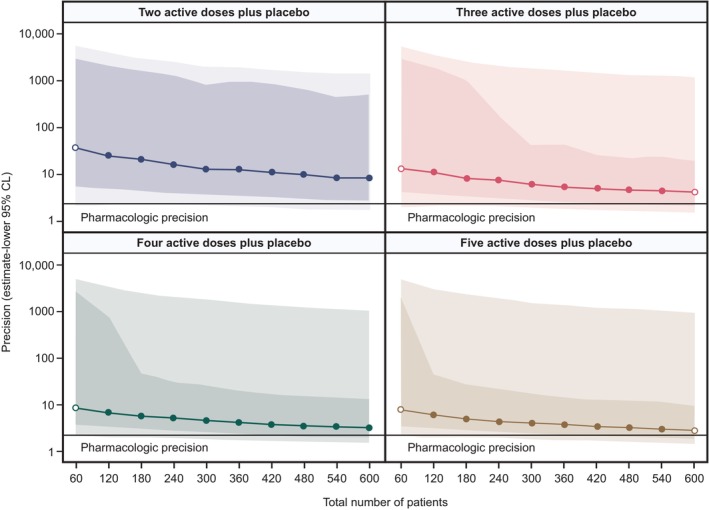
Estimated dose precision for different efficacy trial designs with estimated pharmacologic dose precision represented. Data are median, IQR (dark color), and IDR (lighter color). Doses were selected at random for an unknown underlying dose–response. CL, confidence limit; IDR, interdecile range; IQR, interquartile range.

The generalized linear model estimated that 80% power was achieved with two active treatment arms and 100 patients per treatment arm, while a power of 90% was achieved with 200 patients per arm in a study with two active arms, in agreement with previous clinical study data.^6^, ^7^ With a fixed total sample size (e.g., if *N* = 300, the number of patients per arm can range from 50 to 100 for 5 to 2 active treatment arms, respectively), power decreased with an increasing number of treatments (as the number of patients per arm was decreasing and therefore the treatment effect is diluted by the addition of lower doses [pairwise comparison]). As expected, 90% power was not reached with a large sample of 200 patients per arm in studies with more than two active arms. By contrast, the nonlinear dose–response model (which combines information from all treatment arms) indicated increasing power to detect the *E*_max_ and ED_90_ with increasing number of active treatments and patients. A total trial population of 300 patients was estimated to provide 35–55% power to detect the ED_90_. The probability of achieving precision as high as that established with blood eosinophil pharmacology was, however, < 3% across the designs evaluated (**Figure** **3**).

### Simulations based on BEC reduction for CRSwNP, EGPA, and HES Phase III dose selection

The mepolizumab population PK/PD model was informed by 29,575 blood eosinophil measurements from 2516 participants with various eosinophilic diseases and healthy participants. A total of 2830, 2023, and 2909 blood eosinophil measurements were included from 205 CRSwNP, 135 EGPA, and 192 HES patients, respectively. The depemokimab population PK/PD model was informed by the mepolizumab model, and by 675 blood eosinophil measurements from 48 patients with mild‐to‐moderate asthma from the depemokimab Phase I FTiH asthma trial. Final population PK/PD model parameters are shown in **Table** **2**; details relating to model fit are shown in **Supplementary Information and Figures** **S2–S6**.

**Table 2:** Final population PK/PD parameter estimates for depemokimab

Parameter	Theta	SE	%RSE^a^	Parameter estimate (95% CI)^b^
KRO (GI/L)	−1.73	0.0529	3.06	0.177 (0.160, 0.197)
KOUT (1/h)	−4.35	0.0921	2.12	0.0129 (0.259, 0.371)
IC_50_ (μg/mL)	−2.34	0.0789	3.37	0.0960 (0.0822, 0.112)
*I*_max_	−1.77	0.0265	1.50	0.170 (0.162, 0.179)
GAMA	1.61	0.0559	3.47	1.61 (1.50, 1.72)
BEOS2 on *I*_max_	−0.380	0.0339	8.92	−0.380 (−0.446, −0.314)
BEOS1 on KRO	0.563	0.0226	4.01	0.563 (0.519, 0.607)
SA KRO	0.438	0.0546	12.5	0.438 (0.331, 0.545)
CRSwNP KRO	0.132	0	0	0.132 (0.132, 0.132)
EGPA KRO	0.208	0	0	0.208 (0.208, 0.208)
HES KRO	1.27	0.103	8.11	1.27 (1.068, 1.472)
Mepolizumab (Study 200622(8)) HES KRO	0.5	0	0	0.5 (0.5, 0.5)
**Variability**	**OMEGA** ^**2**^	**SE**	**%RSE** a	**%CV** c
BPV KRO	0.0853	0.0161	18.9	29.8
BPV *I*_max_	0.294	0.0866	29.5	58.5
	**SD**	**SE**	**%RSE** a	
Residual error	0.350	0.0203	5.81	
**PD parameter estimation** KROi = EXP(θ_KRO_ + θ_BEOS1_*LOG(BEOS/0.26) + θ_SA_*hasSA + θ_HES_*hasHES + θ_EGPA_*hasEGPA + +θ_CRSwNP_*hasCRSwNP + ETA1)*θ_200622_ KOUTi = 24*EXP(θ_OUT_) IC_50_i = EXP(θ_IC50_) I_maxi_ = EXP(θ_Imax_ + θ_BEOS2_*LOG(BEOS/0.26) + ETA2) GAMAi = θ_GAMA_ ETAi~N(0,OMEGA^2^) **Residual error** ε1 ~ N(0, SD^2^) Y = IPRED*exp(ε1)				

Parameters highlighted in gray were fixed to values estimated from pooled data from 16 mepolizumab studies. BEC, blood eosinophil count; BEOS, BEC at baseline; BPV, between‐patient variability; CI, confidence interval; CRSwNP, chronic rhinosinusitis with nasal polyps; CV, coefficient of variation; ε, residual variability; EGPA, eosinophilic granulomatosis with polyangiitis; HES, hypereosinophilic syndrome; GAMA, Hill slope; i, individual patient parameter; IC_50_, concentration resulting in 50% of maximum effect; *I*_max_, maximum effect; KRO, baseline blood eosinophils; KOUT, rate of elimination; PD, pharmacodynamic; PK, pharmacokinetic; %RSE, percent relative standard error of the estimate; SA, severe asthma; SD, standard deviation; SE, standard error.

^a^ %RSE was calculated as (SE/parameter estimate)*100.

^b^ 95% CI was calculated as Theta ± 1.96*SE and back‐transformed for back‐transformed parameters.

^c^ %CV is calculated as sqrt(exp(OMEGA)‐1)*100 for exponential error model.

**Figure 4:**
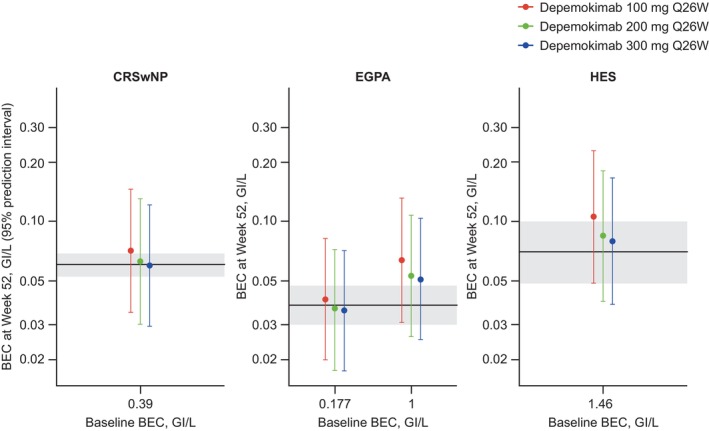
Predicted geometric mean (95% prediction interval) of BEC at end of treatment (Week 52), for different depemokimab dosing regimens for CRSwNP, EGPA, and HES. All predictions are based on no dose delay. The horizontal black line and gray shaded area in each graph represent the geometric mean BEC (95% CI) at end of mepolizumab treatment in the reference studies (SYNAPSE, MIRRA, and GSK 200622, respectively). BEC, blood eosinophil count; CI, confidence interval; CRSwNP, chronic rhinosinusitis with nasal polyps; EGPA, eosinophilic granulomatosis with polyangiitis; HES, hypereosinophilic syndrome; Q26W, every 26 weeks.

For CRSwNP, depemokimab 100 mg SC Q26W was predicted to reduce BEC from baseline by 82% for the entire trial duration, comparable to mepolizumab 100 mg SC Q4W in the reference trial SYNAPSE (84% reduction; **Figure** **4**).^5^ For EGPA and HES, depemokimab 200 mg SC Q26W was predicted to reduce BEC from baseline by ≥ 79% and 94%, respectively, for the entire trial duration, comparable to the effect of mepolizumab 300 mg SC Q4W in the reference trials MIRRA and GSK 200622 (79% and 95% reduction, respectively; **Figure** **4**).^8^, ^10^ Furthermore, for EGPA, depemokimab 200 mg SC Q26W was predicted to reduce blood eosinophils close to the target range observed with mepolizumab when baseline BEC is 1000 cells/μL, which is considered more indicative of the “true” baseline blood eosinophils when OCS are tapered, with a visible advantage compared with 100 mg SC Q26W in terms of blood eosinophil reduction (**Figure** **4**). In all three diseases, there was limited additional benefit in terms of blood eosinophil reduction at higher doses. At doses of 100 and 200 mg SC Q26W (i.e., twice‐yearly), depemokimab provides a trough plasma concentration five and 10 times the EC_50_, respectively.

## DISCUSSION

When an abundance of high‐quality safety and efficacy data is available for an established mechanism of action where MVs and TVs for modulation of a pharmacologic endpoint of approved efficacy have been demonstrated, the combination of MIDD and QDM has the potential to markedly shorten clinical development.^42^ For the current example of depemokimab, the use of clinical trial simulations founded on past clinical experience highlighted that conducting a conventional efficacy‐based Phase IIb dose‐ranging study using the clinical endpoint of exacerbation would not support dose selection for the Phase III program in severe asthma. In fact, the precision offered for identification of clinical dose was vastly lower than that of the well‐characterized pharmacologic endpoint of blood eosinophil reduction. These methodologies supported the omission of a Phase IIb study, thereby shortening the development of depemokimab by approximately 2–3 years.

This shortened development path of depemokimab was made possible due to existing knowledge of (1) the common mechanism and site of action of IL‐5 therapies, including BEC reductions (a marker to match pharmacology) with the anti‐IL‐5 treatment mepolizumab from two Phase III studies in patients with severe asthma^6^, ^9^; (2) robust BEC data collected during the washout phase of the Phase I depemokimab trial in a relevant population (mild‐to‐moderate asthma),^18^ allowing translation to severe asthma; (3) the use of exposure–response models to describe the mepolizumab and depemokimab exposure–BEC relationship across different diseases; and (4) the fact that targeting IL‐5 has a well‐established and acceptable safety profile for the specific biologics developed and tested so far.^6^, ^43^, ^44^ Clinical trial simulations have the advantage of being able to explore a wide range of plausible trial designs and a wide dose range. The Phase IIb trial simulation results indicated that, as expected for a fixed overall trial size, study power to detect treatment effects decreases (pairwise comparison) with increasing numbers of treatment arms due to the number of patients per arm decreasing and therefore dilution of the treatment effect by lower doses. Moreover, the probability that any trial could estimate the clinical dose (defined as the dose producing 90% of maximal effect) with better precision than the pharmacologic reference value was < 3%. These results illustrated the general failure to identify dose–response in Phase IIb without the use of low doses and appropriate dose–response models, and the inherent challenge and ultimate futility of selecting doses using conventional efficacy endpoints in Phase II for depemokimab.

A Phase III depemokimab dose for patients with severe asthma and elevated BEC was selected based on the precedented blood eosinophil pharmacologic endpoint and determination of the probability of achieving specific reductions known to translate into efficacy. The results suggested, with high precision, that depemokimab 100 mg SC Q26W would provide a robust dosing regimen that confers the same level of efficacious pharmacology to mepolizumab in Phase III trials.^6^, ^9^ A simulation approach was also used to select depemokimab doses for the Phase III program in CRSwNP (100 mg SC Q26W), EGPA (200 mg SC Q26W), and HES (200 mg SC Q26W). EGPA and HES are rare diseases, with incidence ranging from 0.5 to 4.2 cases (EGPA) or 1.6 to 3.6 cases (HES) per million people per year,^45^, ^46^ rendering the conduct of conventional dose‐ranging studies extremely challenging. This further supported the use of a pharmacologic endpoint (BEC) for dose selection for pivotal Phase III trials, and the use of MIDD methodology to select a dose, not previously tested in a clinical trial, with confidence.

Conventional efficacy measures such as clinical exacerbation rate, which are used commonly for severe asthma, have sufficient uncertainty to make active treatment comparisons and identification of the dose–response troublesome in Phase II due to sample size. However, the use of a single measure such as BEC to predict efficacy in CRSwNP should be carefully considered, as the relationship between clinical efficacy and BEC is currently less clearly defined in CRSwNP than in severe asthma. This strategy, however, was also used to inform mepolizumab dose selection across indications based on similar translational considerations.^47^ Additionally, as noted above, Phase IIb trials are not feasible for rare diseases such as EGPA and HES due to limited patient populations. While there is less clinical evidence available for EGPA and HES, both diseases are characterized by excess blood eosinophil production; therefore, strategies aimed at reducing BEC, including anti‐IL5 therapies, are expected to be of clinical benefit in these patient populations.^48^ Differences in disease pathology and mechanisms of action between biologics may also contribute to variability in treatment outcomes. It should be noted that benralizumab has a different mechanism of action to mepolizumab and depemokimab, as it targets the IL‐5R and requires recruitment of other cell types and/or complement to deplete eosinophils.^44^ Although blood eosinophils are likely to be relevant to bridge pharmacology across molecules with a similar mechanism of action (e.g., mepolizumab and depemokimab), such an approach may not translate between different mechanisms of action (e.g., mepolizumab and benralizumab).

The adequacy of the MIDD and QDM approach is supported by results from Phase III studies of depemokimab in patients with severe asthma and CRSwNP with underlying type 2 inflammation.^15^, ^16^ In the Phase III SWIFT‐1 and SWIFT‐2 asthma trials, depemokimab 100 mg SC Q26W reduced BEC by 80% from baseline at Week 2 (pooled population), and these remained reduced throughout the trials and at Week 52 (82% reduction from baseline). In the replicate Phase III ANCHOR‐1 and ANCHOR‐2 studies in patients with CRSwNP, depemokimab (100 mg SC Q26W) significantly reduced BEC from the first timepoint assessed (Week 4), which was maintained throughout the 52‐week study (ratios to baseline vs. placebo: 85% across both studies [*P* < 0.001]).^16^ Importantly, the BEC reductions were accompanied by clinical efficacy in the SWIFT and ANCHOR studies, with significant improvements in the primary endpoints vs. placebo.

This study has several limitations. Extrapolation from asthma to other eosinophilic diseases (CRSwNP, EGPA, and HES) is inherently challenging due to potential differences in exposure–response dynamics and disease pathophysiology. To address this, separate modeling approaches were employed: the Bayesian dose–time response model supported dose selection in asthma, while a mechanistic PK/PD model, informed by the mepolizumab clinical program, was used for CRSwNP, EGPA, and HES. Leveraging the mepolizumab PK/PD meta‐analysis, which included a broad range of disease population, severities, and exposure–BEC dynamics,^47^ helped ensure that extrapolation assumptions were data‐driven. In addition, QDM thresholds for BEC suppression were predefined based on the steady‐state eosinophil reductions previously achieved with mepolizumab, at which clinical efficacy was established. This strategy was grounded in pharmacology matching, whereby the BEC targets for depemokimab were directly aligned to those observed with mepolizumab, without making explicit assumptions about the quantitative relationship between BEC reduction and clinical efficacy. Furthermore, the presented simulations of dose‐ranging trials were based on Phase II trials using the mepolizumab‐precedented exacerbation rate as the sole efficacy endpoint, consistent with DREAM and other clinical trials.^6^, ^7^, ^9^ While other endpoints (e.g., symptom score, patient‐reported outcomes, or spirometry) might offer additional insights into dose differentiation, such analyses are limited by variability in reported outcomes across Phase II/III asthma studies. Also, it should be noted that, like exacerbations, these endpoints are associated with considerable between‐patient variability, and their relationship with exacerbations, the key registrational endpoint, remains unclear. Finally, the analysis provided no assessment of the depemokimab safety profile. Mepolizumab has demonstrated long‐term tolerability,^12^, ^13^, ^14^ and although depemokimab includes a YTE modification, this change was not expected to substantially alter the safety profile.^49^ Consistent with this, Phase I and III studies in patients with severe asthma or CRSwNP have demonstrated that depemokimab is well tolerated, with safety followed for up to 2 years of depemokimab treatment in patients with severe asthma.^15^, ^16^, ^18^, ^50^

### Conclusions

This case study demonstrates how MIDD can streamline drug development, bring new medicines to patients faster, and improve R&D productivity. When conditions allow, such accelerated development can have significant implications, ensuring patients receive timely access to new, effective therapies with less frequent dosing.

## FUNDING

The sponsor (GSK) funded the modeling/analysis presented in this paper and also the original studies on which the analysis was based. The sponsor was involved in study design and implementation, as well as data collection, analysis, interpretation, writing the study reports and reviewing this manuscript. The sponsor did not place any restrictions on access to data or statements made in the manuscript. All authors had full access to the data upon request and had final responsibility for the decision to submit for publication.

## CONFLICT OF INTEREST

C.Z., S.S., A.B., N.B., R.F., and D.A. are employees of GSK and hold financial equities in the company. I.J.P. is a former employee of GSK and holds financial equities in the company.

## AUTHOR CONTRIBUTIONS

All authors wrote the manuscript. I.J.P., N.B., R.F., and D.A. designed and performed the research. C.Z., S.S., I.J.P., A.B., N.B., R.F., and D.A. analyzed the data.

## Supporting information


Figure S1.

## Data Availability

Please refer to GSK weblink to access GSK’s data sharing policies and as applicable seek anonymized subject level data via the link https://www.gsk‐studyregister.com/en/.
