# Supplementary material for: Straight to Phase III: Model‐Informed Approach Speeds Depemokimab Clinical Development in Interleukin‐5‐Driven Diseases
Source: Clin Pharmacol Ther. 2026 Jan 20;119(4):1005–15. doi: 10.1002/cpt.70183 (PMC12997505; doi:10.1002/cpt.70183)
Supplement: Supplementary file 1 — Figure S1. [file CPT-119-1005-s001.docx]

# Running title: Model-informed Approach Speeds Depemokimab Development

# Straight to Phase III: Model-informed approach speeds depemokimab clinical development in interleukin-5-driven diseases

Chiara Zecchin,^1^ Stein Schalkwijk,^2^ Isabelle J Pouliquen,^3^*** Alienor Berges,^4^ Nicholas Bird,^5^
Richard Follows,^6^ Daren Austin^7^

*^1^Clinical Pharmacology Modelling and Simulation, GSK, Stevenage, UK;* *^2^GSK, Amsterdam, Netherlands; ^3^Clinical Pharmacology Modelling and Simulation, GSK, London, UK; ^4^Clinical Pharmacology, GSK, Stevenage, UK; ^5^Biostatistics, GSK, London, UK; ^6^Clinical Sciences – Respiratory, Immunology & Inflammation Unit (RIIRU), GSK, London, UK; ^7^Business Development, GSK, London, UK*

**At the time of the study*

**Corresponding author:** Chiara Zecchin

**Address:** Clinical Pharmacology Modelling and Simulation, GSK, Gunnels Wood Road, Stevenage,
SG1 2NFX, UK

**Conflict of interest**

**C.Z.**, **S.S.**, **A.B.**, **N.B.**, **R.F.**, and **D.A.** are employees of GSK and hold financial equities in the company. **I.J.P.** is a former employee of GSK and holds financial equities in the company.

**Funding**

The sponsor (GSK) funded the modeling/analysis presented in this paper and also the original studies on which the analysis was based. The sponsor was involved in study design and implementation, as well as data collection, analysis, interpretation, writing the study reports and reviewing this manuscript. The sponsor did not place any restrictions on access to data or statements made in the manuscript. All authors had full access to the data upon request and had final responsibility for the decision to submit for publication.

**Keywords (3-10 max):** Biologics, Dose, Estimation methods, Immunology, Model-based drug development, Pharmacokinetics, Pharmacokinetics-pharmacodynamics, Pharmacometrics

**Target Journal:** *CPT*

**Article type:** Article (substantial novel original research)

# SUPPLEMENTARY INFORMATION

## Supplementary Methods

The indirect response model developed on meta-analysis data from 16 mepolizumab studies^1–16^ was used. Pharmacokinetic and half-maximal inhibitory concentration (IC_50_) parameters were re-estimated (maintaining the same model structure), based on the data from the Phase I Study 205722 to reflect the longer half-life and enhanced potency of depemokimab compared with mepolizumab. The model (Equation 1) describes the effect of depemokimab concentration (C) on eosinophils (eos) and is parameterized in terms of baseline eos (KRO), rate of elimination (KOUT), maximum inhibitory effect (Imax), IC_50_, and Hill slope (GAMA). Depemokimab is assumed to reduce eos production (as determined for mepolizumab) and a GAMA was included in the model and fixed to the value estimated for mepolizumab. The effect of covariates on model parameters was assumed to be the same determined for mepolizumab. Measured baseline blood eosinophil count is a covariate of KRO and Imax. Disease and Study 200622 were covariates of KRO.

## Figure S1. Probability of exceeding the MENSA and MUSCA pharmacology based on QDM principles (Black & White version)


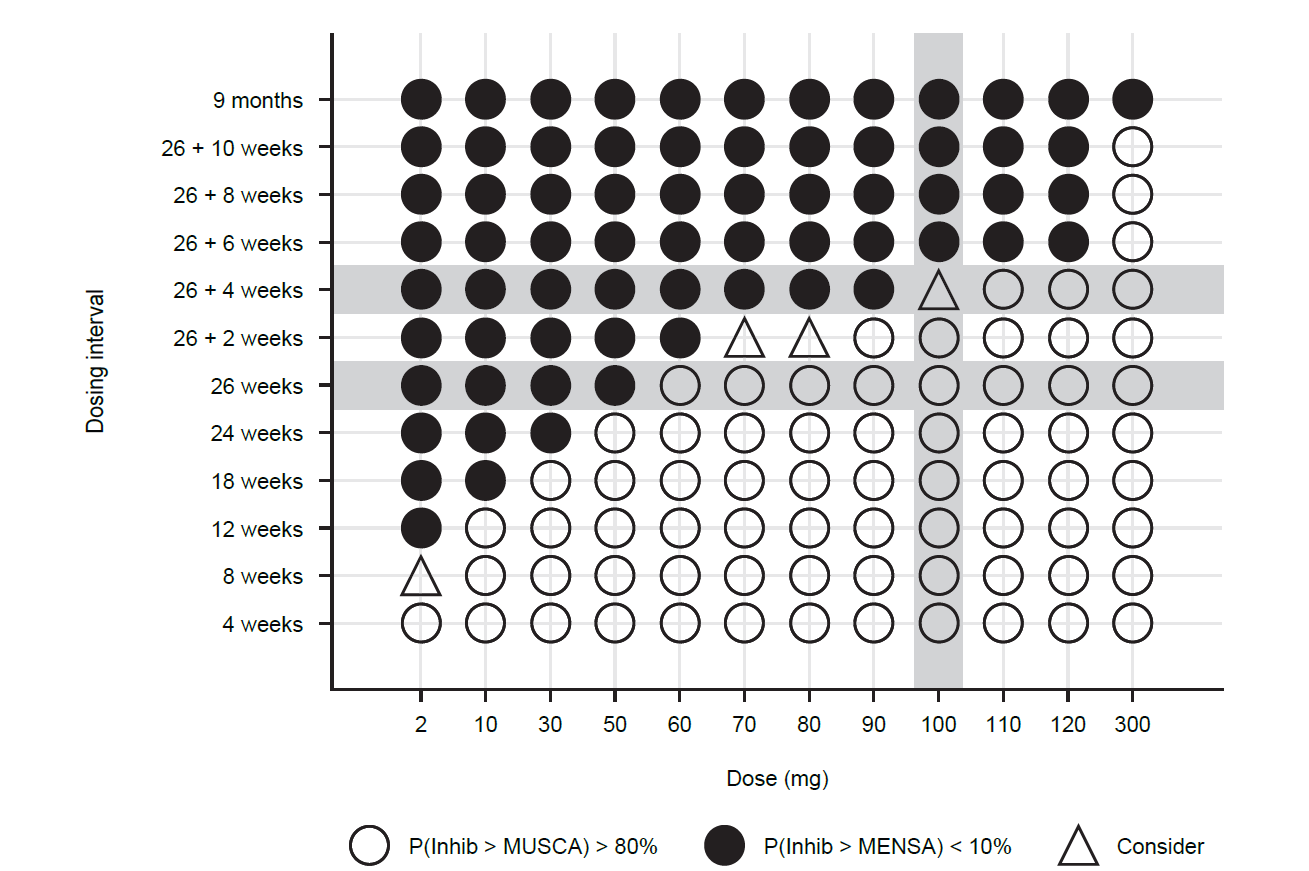


Prespecified QDM go and no-go criteria were applied to the 12 doses and 12 dosing intervals. A regimen was deemed a “go” provided the probability of exceeding the MV was >80% (white), and a regimen was deemed a “no go” if the probability of exceeding the TV was <10% (black). Anything falling between these values was deemed a “consider” (gray).

QDM, quantitative decision-making; MV, minimum value; TV, target value.

## Figure S2. Prediction-corrected visual predictive check of mepolizumab meta-analysis PK/PD model (initial model, based on 13 studies across various diseases and healthy volunteers)


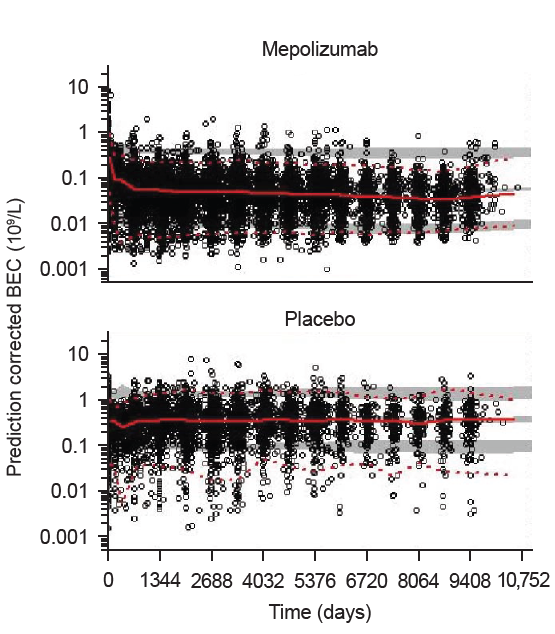


The solid red line represents the median of the prediction corrected observed BEC, the dashed red lines represent the 5% and 95% percentiles of the prediction corrected observed BEC. The black dots represent the prediction corrected observations. The gray shaded areas represent the 95% CI of, from bottom to top, the 5^th^, 50^th^ (i.e., median), and 95^th^ model predicted percentiles.

BEC, blood eosinophil count; CI, confidence interval; PD, pharmacodynamic; PK, pharmacokinetic.

## Figure S3. Visual predictive check of mepolizumab meta-analysis PK/PD model (updated with data from Study 200622) versus BEC data from Phase III study in HES (Study 200622) – BEC absolute values (GI/L)
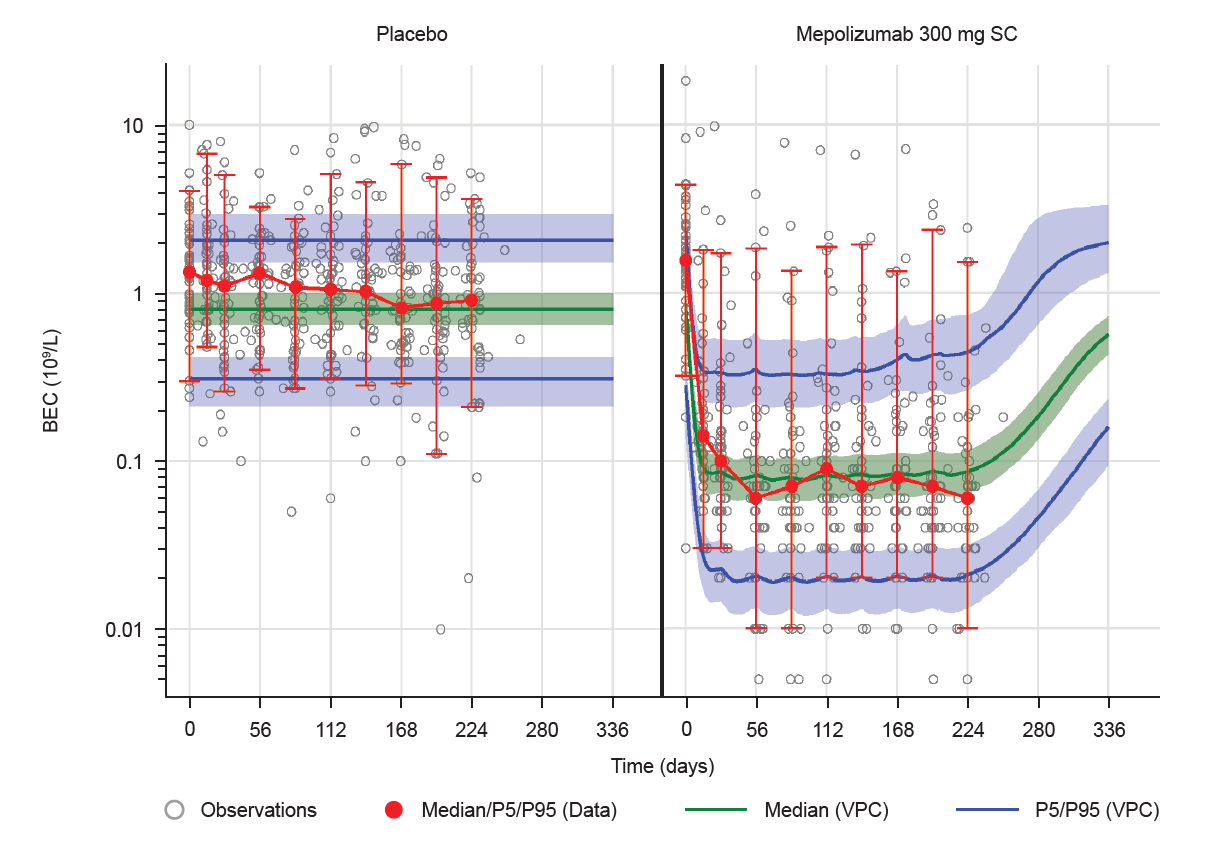


The solid red line represents the median of the observed BEC, the red error bars represent the 5% and 95% percentiles of the observed BEC. The black dots represent the observations. The green shaded areas represent the 95% CI of the model predicted median, the blue shaded area represent the 95% CI of the model predicted 5^th^ and 95^th^ percentiles.

BEC, blood eosinophil count; CI, confidence interval; HES, hypereosinophilic syndrome;
KRO, baseline blood eosinophils; PD, pharmacodynamic; PK, pharmacokinetic; SC, subcutaneous; VPC, visual predictive check.

## Figure S4. Visual predictive check of mepolizumab meta-analysis PK/PD model (updated with data from Study MEA115921) versus BEC data from Phase III study in EGPA (Study MEA115921) – BEC absolute values (GI/L)


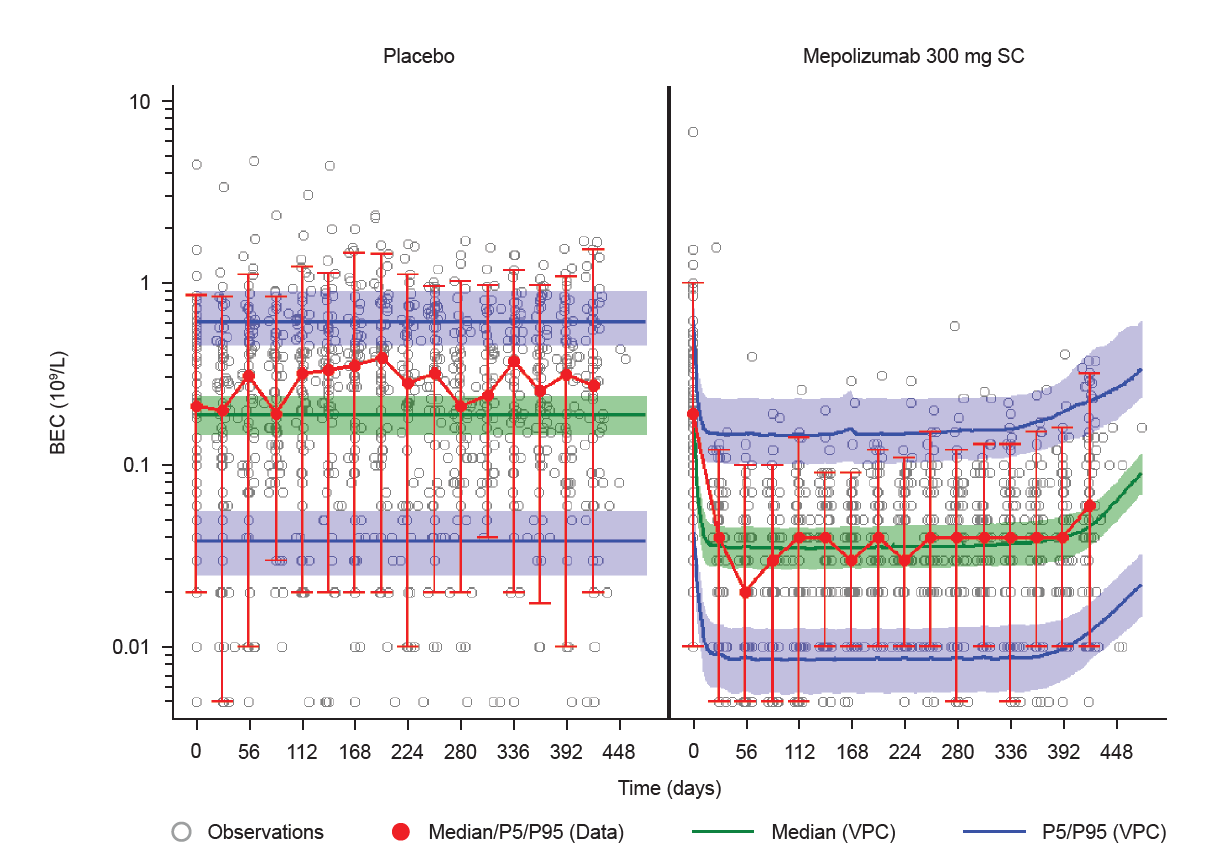


The solid red line represents the median of the observed BEC, the red error bars represent the 5% and 95% percentiles of the observed BEC. The black dots represent the observations. The green shaded areas represent the 95% CI of the model predicted median, the blue shaded area represent the 95% CI of the model predicted 5^th^ and 95^th^ percentiles.

BEC, blood eosinophil count; CI, confidence interval; EGPA, eosinophilic granulomatosis with polyangiitis; PD, pharmacodynamic; PK, pharmacokinetic; SC, subcutaneous; VPC, visual predictive check.

## Figure S5. Visual predictive check of mepolizumab meta-analysis PK/PD model (updated with data from Study 205687) versus BEC data from Phase III study in CRSwNP (Study 205687) – BEC absolute values (GI/L)


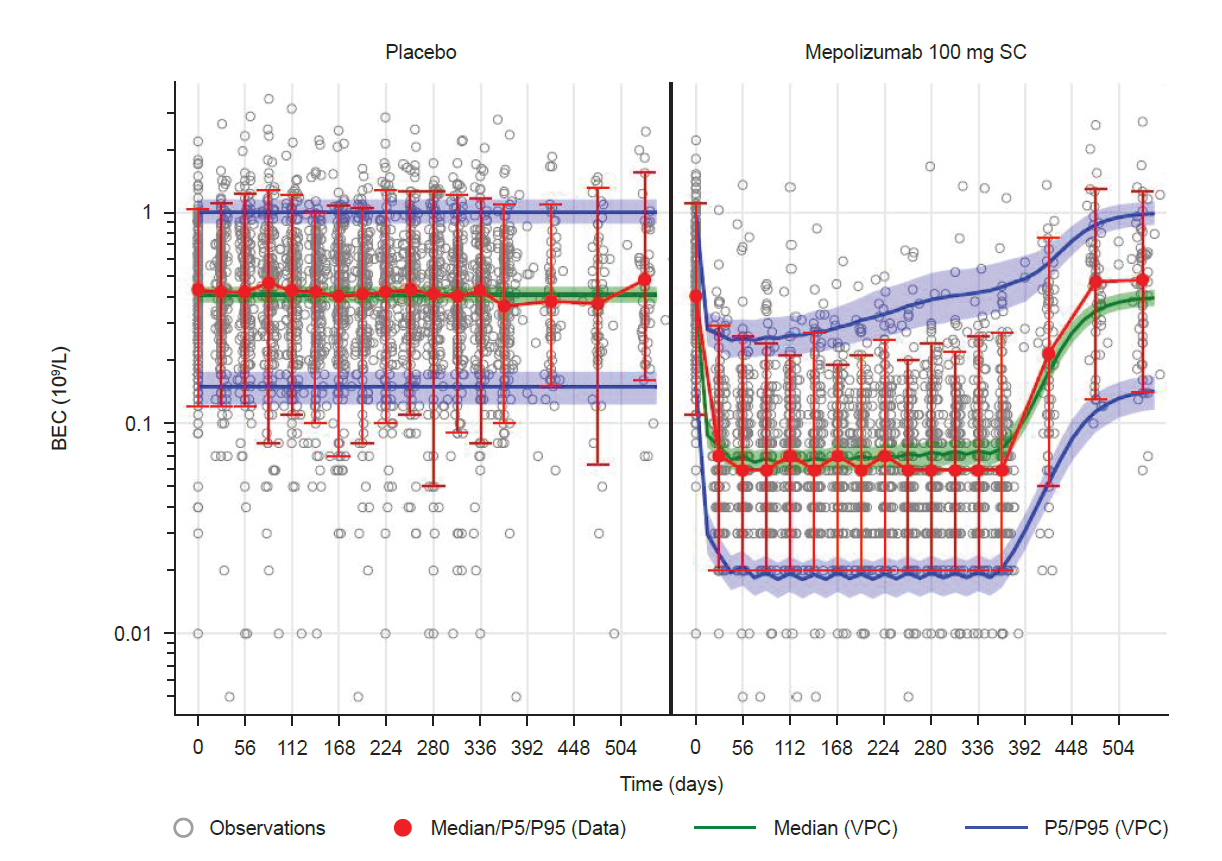


The solid red line represents the median of the observed BEC, the red error bars represent the 5% and 95% percentiles of the observed BEC. The black dots represent the observations. The green shaded areas represent the 95% CI of the model predicted median, the blue shaded area represent the 95% CI of the model predicted 5^th^ and 95^th^ percentiles.

BEC, blood eosinophil count; CI, confidence interval; CRSwNP chronic rhinosinusitis with nasal polyps; PD, pharmacodynamic; PK, pharmacokinetic; SC, subcutaneous; VPC, visual predictive check.

## Figure S6. Visual predictive check of meta-analysis PK/PD model (updated with data from depemokimab FTIH Study 205722) versus BEC data from depemokimab FTIH study (Study 205722)


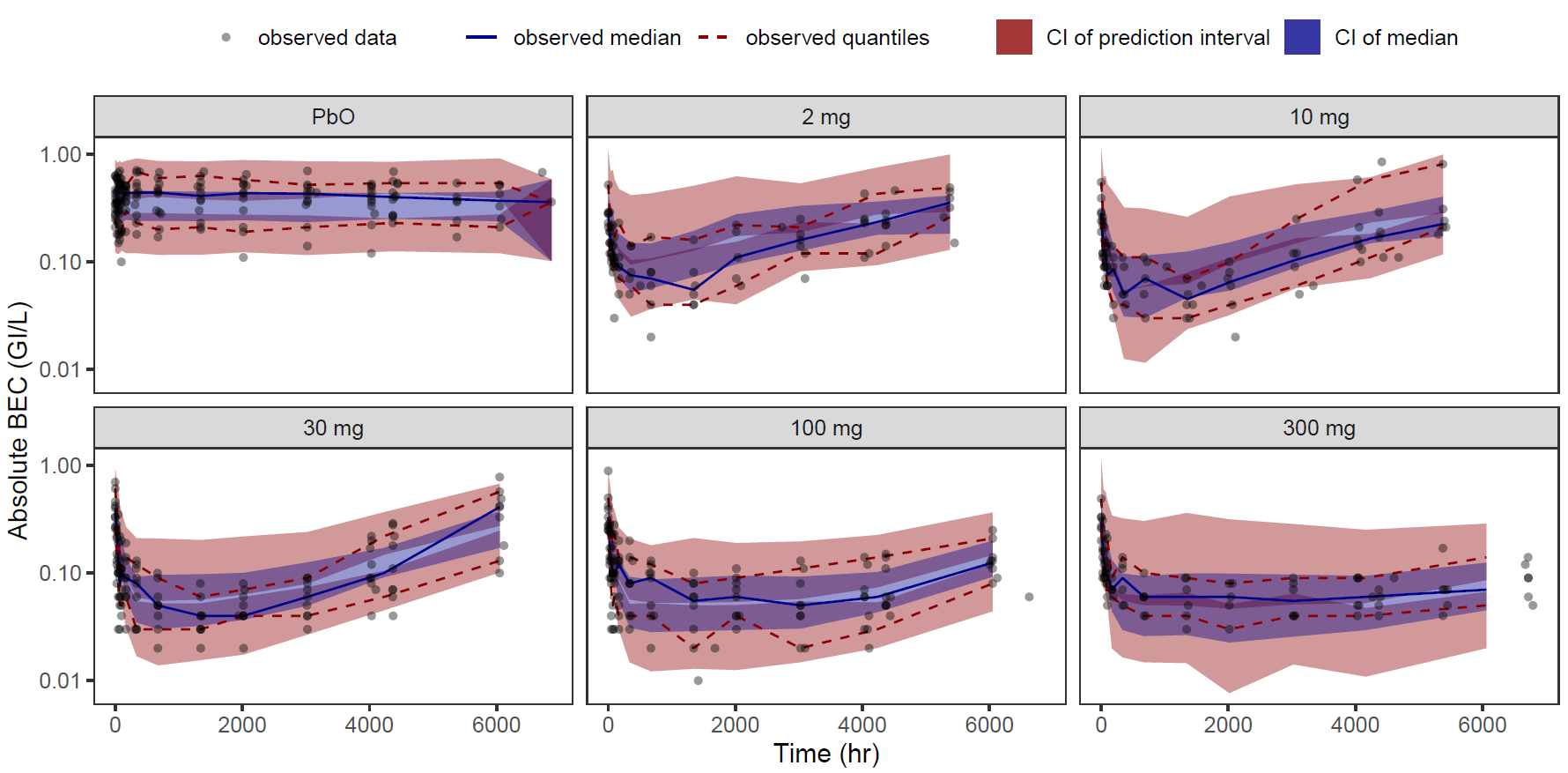


The solid blue line represents the median of the observed BEC, the red dashed lines represent the 5% and 95% percentiles of the observed BEC. The black dots represent the observations. The blue shaded areas represent the 95% CI of the model predicted median, the red shaded area represent the 95% CI of the model predicted 5th and 95th percentiles.

BEC, blood eosinophil count; CI, confidence interval; FTiH, first-time-in-human; PD, pharmacodynamic; PK, pharmacokinetic; Pbo, placebo; SC, subcutaneous; VPC, visual predictive check.

# SUPPLEMENTARY REFERENCES

1. Han, J.K. *et al.* Mepolizumab for chronic rhinosinusitis with nasal polyps (SYNAPSE): a randomised, double-blind, placebo-controlled, phase 3 trial. *The Lancet. Respiratory medicine* **9**, 1141-1153 (2021).
2. Roufosse, F. *et al.* Efficacy and safety of mepolizumab in hypereosinophilic syndrome: A phase III, randomized, placebo-controlled trial. *The Journal of allergy and clinical immunology* **146**, 1397-1405 (2020).
3. Wechsler, M.E. *et al.* Mepolizumab or Placebo for Eosinophilic Granulomatosis with Polyangiitis. *The New England journal of medicine* **376**, 1921-1932 (2017).
4. GSK. Study SB-240563/001 (Study 001); Phase 2a single dose, dose-ranging PK, PD and safety study in male patients with asthma. (1998).
5. GSK. Study SB-240563/035 (Study 035); Phase 1 single dose, dose-ranging PK, PD and safety study in male patients with asthma. (1998).
6. GSK. Study SB-240563/006 (Study 006); Phase 2 PK, three monthly-dose, safety and efficacy study in patients with asthma. (1999).
7. GSK. Study SB-240563/017 (Study 017); Phase 2, three-dose, PK, PD and safety study in patients with asthma. (2000).
8. GSK. Study SB-240563/018 (Study 018); Phase 1 PK, bioavailability single-dose study in healthy volunteers. (2001).
9. GSK. MHE100185; A Multicenter, Randomized, Double-blind, Placebo-controlled, Parallel Group Phase III Study to Evaluate Corticosteroid-reduction and -sparing Effects of Mepolizumab 750 mg Intravenous in Subjects With Hypereosinophilic Syndromes (HES) and Evaluate Efficacy and Safety of Mepolizumab in Controlling the Clinical Signs and Symptoms of Subjects With HES. (2006).
10. GSK. MEE103226; A Randomised, Double-Blind, Placebo-Controlled, Single-Centre Study to Provide a Preliminary evaluation of Pharmacokinetics, Pharmacodynamics, Safety and Tolerability of Intravenous anti-human interleukin-5 (mepolizumab, 750mg and 1500mg) in the treatment of Eosinophilic Oesophagitis in Adults. (2007).
11. GSK. MEE103219; A randomized, double-blind, parallel group clinical trial to assess safety, tolerability, pharmacokinetics, and pharmacodynamics of intravenous mepolizumab (SB240563)(0.55mg/kg, 2.5mg/kg or 10mg/kg) in pediatric subjects with eosinophilic esophagitis, aged 2 to 17 years (2008).
12. GSK. MEA112997; A multicenter, randomized, double-blind, placebo-controlled, parallel group, dose ranging study to determine the effect of mepolizumab on exacerbation rates in subjects with severe uncontrolled refractory asthma. (2012).
13. GSK. MEA114092; A multicenter, open-label, dose ranging study to determine the pharmacokinetics and pharmacodynamics of mepolizumab administered intravenously or subcutaneously to adult asthmatic subjects with elevated blood eosinophil levels. (2012).
14. GSK. MEA115705; A single blind, placebo controlled, parallel group, single ascending intravenous dose study to assess safety, tolerability, pharmacokinetics and pharmacodynamics of SB-240563 (mepolizumab) in healthy Japanese male subjects. (2012).
15. GSK. MEA115575; A Randomised, Double-Blind, Placebo-Controlled, Parallel-Group, Multicenter Study of Mepolizumab Adjunctive Therapy to Reduce Steroid Use in Subjects with Severe Refractory Asthma. (2013).
16. GSK. MEA115588; A randomised, double-blind, double-dummy, placebo-controlled, parallel-group, multi-centre study of the efficacy and safety of mepolizumab adjunctive therapy in subjects with severe uncontrolled refractory asthma. (2014).
